# American Dental Society of Europe

**Published:** 1899-05

**Authors:** 


### American Dental Society of Europe.

At the last meeting the following action was taken and is self-explanatory. Upon motion, the following report of the committee appointed by the American Dental Society of Europe in London, August, 1898, was carried unanimously:

WHEREAS, A Special Executive Session of the American Dental Society of Europe has been called at Brussels, April 1, 1899, for the purpose of considering what further action shall be taken towards improving the standing of the graduates from American Dental Colleges practising in Europe and to receive and act upon the report of the committee appointed in London at the last annual meeting,

WHEREAS, a majority of said committee and a large number of the active members of the society being present from all parts of Europe, thereby showing their great interest in the subject under consideration,

*Resolved*, "That the American Dental Society of Europe views with pleasure and approval the action of the National Association of Dental Faculties, U. S. A., in appointing a 'Foreign Relations Committee,' and hopes that this committee will be indefinitely continued, and empowered to take such action as shall appear to its members to be for the best interests of the profession."

*Resolved*, "That the society expresses its thanks to the National Association of Dental Faculties, for its resolution and action in accepting the report of its 'Foreign Relations Committee,' and continuing it at Omaha, August, 1898, and creating Advisory Boards in all European countries, with the view that the certificates of foreign students proposing to enter American dental colleges, be submitted to the Advisory Boards of the respective countries of which they are citizens or residents."

*Resolved*, "That it is the opinion of this society, that foreign students should possess such a knowledge of the English language as will enable them to thoroughly comprehend the lectures and teachings which they will be called upon to pass examinations in, and that no foreign student should be allowed to pass any examination through the medium of an interpreter."

*Resolved*, "That the American Dental Society of Europe heartily approves of the wisdom of requiring a preliminary examination of students from European countries, or would suggest as preferable that it be required of each foreign student that he present official certificates of having passed the preliminary requirements for matriculation as a dental student in his own country, and that these certificates be endorsed by the Advisory Board of said country, and that they also be subject to the rules of the National Association of Dental Faculties."

*Resolved*, "That this society approves of the resolution of the 'Foreign Relations Committee,' of the National Association of Dental Faculties, to appoint Advisory Boards consisting of 'not more than three members,' and it is hoped, for the accomplishment of the best results, that the number of members on each board be raised to three at the earliest practical moment, and this society is unanimously and strongly of the opinion, that three are necessary to constitute an influential board of this nature; and that, where practicable, at least one member should be a native of the country."

*Resolved*, "That the American Dental Society of Europe tender a sincere vote of thanks to the National Association of Dental Faculties, and to the 'Foreign Relations Committee,' and especially to their energetic chairman, Dr. W. C. Barrett, for the active and hearty manner in which they have met the appeals of their confreres in Europe, who for so long have urged the importance of the consideration this subject is now receiving at their hands."

*Resolved*, "That a Committee upon Dental Education be a permanent committee of the society, and that said committee consist of all the members of the society, who are members of the Advisory Board of the 'Foreign Relations Committee' of the National Association of Dental Faculties."

(Signed) L. C. BRYAN, *Chairman.*
W. E. ROYCE.
W. MITCHELL.

It was further moved and carried:—"That the report of the committee be sent to the various dental journals for publication at the earliest possible date."

